# Outer Membrane Vesicles Derived from *Klebsiella pneumoniae* Influence the miRNA Expression Profile in Human Bronchial Epithelial BEAS-2B Cells

**DOI:** 10.3390/microorganisms8121985

**Published:** 2020-12-13

**Authors:** Federica Dell’Annunziata, Concetta Paola Ilisso, Carmela Dell’Aversana, Giuseppe Greco, Alessandra Coppola, Francesca Martora, Fabrizio Dal Piaz, Giuliana Donadio, Annarita Falanga, Marilena Galdiero, Lucia Altucci, Massimiliano Galdiero, Marina Porcelli, Veronica Folliero, Gianluigi Franci

**Affiliations:** 1 Department of Experimental Medicine, University of Campania Luigi Vanvitelli, 80138 Naples, Italy; federica.dellannunziata@unicampania.it (F.D.); petgiuseppegreco@gmail.com (G.G.); francesca.martora@unicampania.it (F.M.); marilena.galdiero@unicampania.it (M.G.); massimiliano.galdiero@unicampania.it (M.G.); 2 Department of Precision Medicine, University of Campania Luigi Vanvitelli, 80138 Naples, Italy; concettapaola.ilisso@unicampania.it (C.P.I.); carmela.dellaversana@cnr.it (C.D.); alessandra.coppola@unicampania.it (A.C.); lucia.altucci@unicampania.it (L.A.); marina.porcelli@unicampania.it (M.P.); 3 Department of Medicine, Surgery and Dentistry Scuola Medica Salernitana, University of Salerno, 84081 Salerno, Italy; fdalpiaz@unisa.it (F.D.P.); gdonadio@unisa.it (G.D.); 4 Department of Agricultural Science, University of Naples Federico II, 80055 Naples, Italy; annarita.falanga@unina.it

**Keywords:** *Klebsiella pneumoniae*, outer membrane vesicles, miRNA, immune response, target genes

## Abstract

*Klebsiella pneumoniae* is an opportunistic pathogen that causes nosocomial and community-acquired infections. The spread of resistant strains of *K. pneumoniae* represents a growing threat to human health, due to the exhaustion of effective treatments. *K. pneumoniae* releases outer membrane vesicles (OMVs). OMVs are a vehicle for the transport of virulence factors to host cells, causing cell injury. Previous studies have shown changes of gene expression in human bronchial epithelial cells after treatment with *K. pneumoniae* OMVs. These variations in gene expression could be regulated through microRNAs (miRNAs), which participate in several biological mechanisms. Thereafter, miRNA expression profiles in human bronchial epithelial cells were evaluated during infection with standard and clinical *K. pneumoniae* strains. Microarray analysis and RT-qPCR identified the dysregulation of miR-223, hsa-miR-21, hsa-miR-25 and hsa-let-7g miRNA sequences. Target gene prediction revealed the essential role of these miRNAs in the regulation of host immune responses involving NF-ĸB (miR-223), TLR4 (hsa-miR-21), cytokine (hsa-miR-25) and IL-6 (hsa-let-7g miRNA) signalling pathways. The current study provides the first large scale expression profile of miRNAs from lung cells and predicted gene targets, following exposure to *K. pneumoniae* OMVs. Our results suggest the importance of OMVs in the inflammatory response.

## 1. Introduction

*K. pneumoniae* is a significant opportunistic pathogen, mainly associated with hospital-acquired infections [1]. Studies have estimated that it causes 8% of all nosocomial bacterial infections in Europe and in the United States [2,3]. This bacterium is responsible for a broad spectrum of extraintestinal diseases such as sepsis, pneumonia, urinary tract, lungs, abdominal cavity and soft tissue infections [4]. *K. pneumoniae* has important virulence factors, such as lipopolysaccharides, a capsule, adhesins and siderophores, required for its mechanism of colonization, adherence, invasion and to enable the progression of infection [2,5]. In addition, hemolysins, tyrosine kinase, heat-stable enterotoxins and heat-labile exotoxins participate in the pathogenicity [6,7]. *K. pneumoniae* rapidly acquires antibiotic resistance mechanisms making the selection of the appropriate antibiotic treatment more challenging [1,8]. Carbapenem resistance appears to have the greatest impact on the effectiveness of the treatment. The European Centre for Disease Control and Prevention (ECDCP) assumed that 15.2% of *K. pneumoniae* strains are carbapenem resistant in Italy [9,10]. In this scenario, nosocomial *K. pneumoniae* infections reflect a 50% mortality rate if untreated [11,12]. Given the clinical significance of this pathogen, a better understanding of other mechanisms of virulence is fundamental for designing new strategies to treat Klebsiella infections. It is well established that one of the characteristics of Gram-negative bacteria is their ability to form vesicles from the outer membrane, called outer-membrane vesicles (OMVs) [13,14,15]. OMVs are lipid bilayer spherical nanostructures with a diameter of 20–250 nm that are released into the host environment [16,17,18]. The surface of these vesicles is composed of lipopolysaccharide (LPS), phospholipids and outer membrane proteins [19,20]. The vesicular lumen, however, contains periplasmic and cytoplasmic components, including genetic material and virulence factors, such as invasion associated factors, toxins, and immune response modulators [21,22,23]. Thermolabile toxins and cytolysin have been identified in OMVs produced by *Escherichia coli* [24,25,26]. Haemolytic phospholipase C and alkaline phosphates have been detected in the OMVs of *Pseudomonas aeruginosa* [27,28,29]. Keenan et al. have found vacuolating cytotoxin A in the OMVs produced by *Helicobacter pylori* [18,30,31]. Since OMVs consist of toxins and several virulence determinants, it was postulated that the vesicles play a crucial role in bacteria–host interactions [32]. Previously, we demonstrated that OMVs of *K. pneumoniae* induce a strong inflammatory response in human bronchial epithelial cells (BEAS-2B) [13,14,15]. In these cells, OMVs strongly upregulate the expression of genes, encoding cytokines and chemokines [32]. In addition, the effect of the inflammatory cascade leads to pathogen clearance and host homeostasis [33,34,35]. Therefore, understanding cellular and molecular factors in response to the exposure of OMVs could be highly relevant for susceptibility to infection.

MicroRNAs (miRNAs) are small non-coding RNA molecules that are involved in the post-transcriptional regulation of gene expression [36,37]. These molecules are essential in different biological processes, such as development, proliferation, differentiation, cell death and disease [38]. In infected epithelial cells, downregulation of miRNAs increases the expression of cytokines, chemokines, adhesion factors and costimulatory molecules [39,40,41,42]. Little is known about the function of miRNAs in the human bronchial epithelial cells after OMV interaction. Therefore, the aim of our study was to evaluate the miRNA expression changes in BEAS-2 B treated with OMVs produced by standard and two clinical *K. pneumoniae* strains.

## 2. Materials and Methods

### 2.1. Bacterial Strains and Cell Culture

The strains used in the current study were *K. pneumoniae* reference strains (*K. pneumoniae* ATCC 10031), a multi-sensitive clinical strain of *K. pneumoniae* (MS *K. pneumoniae*) and carbapenemase-producing clinical strains of *K. pneumoniae* (KPC-producing *K. pneumoniae*). Identification and susceptibility patterns were detected by matrix assisted laser desorption ionization–time of flight mass spectrometry (Bruker Dal-tonics, Heidelberg, Germany) and Phoenix BD (Becton Dickinson, NJ, USA) systems, respectively, according to the manufacturer’s instructions [43]. BEAS2B cells (ATCC CRL-9609) from human bronchial epithelial tract were used for the treatments. The cells were grown in Dulbecco’s modified Eagle medium with 10% fetal bovine serum, 1% L-glutamine, 100 U/mL penicillin, 100 µg/mL streptomycin (Gibco BRL, Grand Island, NE, USA) at 37 °C in 5% CO_2_, according to the manufacturer’s instructions. 

### 2.2. OMV Purification

OMV purification was performed following the protocol used by Martora et al. with some slight modifications [13]. Briefly, three *K. pneumoniae* strains were grown in LB broth (600 mL, 37 °C, 180 rpm) to an OD_600_ nm value of 1. The bacterial cells were decanted through centrifugation and supernatants were filtered at 0.45 µm and 0.22 µm (Millex-GS filters, Millipore, Darmstadt, Germany). The cell-free supernatants were centrifuged at 100,000× *g* for 2 h at 4 °C (centrifuge Optima XPN-100 Beckman Coulter and rotor 70Ti, Palo Alto, CA, USA). The pellets were washed in sterile PBS1X by ultracentrifugation and re-suspended in 200 µL of PBS1X. The sterility of the OMVs was checked on LB agar plates. The purified OMVs were stored at −20°C after dynamic light scattering (DLS) analysis.

### 2.3. OMV Characterization

The Z-average size (Z-ave) and polydispersity index (PDI) of the OMVs were defined by DLS. The vesicles were analysed using Malvern Zetasizer ZS90 (Malvern Panalytical Ltd., Malvern, UK). Z-ave defines the mean diameter of the vesicles in nm (d.nm) while PDI describes the particle size distribution. For DLS measurements, 40 µL vesicle aliquots were transferred into disposable cuvettes and gently mixed to provide a homogeneous solution. Three independent aliquots were investigated, and three measurements were made for each. Data were analysed via Dispersion Technology Software (DTS) (V7.01) provided by Malvern Zetasizer Nano-ZS for particle sizing in solution. This software provided the Z-ave and PDI.PDI values lower than 0.05 indicate samples with highly monodisperse vesicular distribution. In contrast, PDI values greater than 0.2 denote samples with very wide vesicular distribution. 

### 2.4. Sodium Dodecyl Sulphate Poly-Acrylamide Gel Electrophoresis (SDS-PAGE)

For protein quantization, vesicles were lysed with a 1% Triton X-100 solution for 1 h at 4 °C. The lysate was centrifuged for 30 min at 14,000× *g*. OMV proteins were quantified by a Bradford assay (HIMEDIA, Einhausen, Germany). Vesicle proteins from *K. pneumoniae* ATCC 10031, MS *K. pneumoniae* and KPC-producing *K. pneumoniae* were analysed by 10% SDS-PAGE. The gel was stained with Coomassie brilliant blue (HIMEDIA, Einhausen, Germany). Proteins in the range of 30–40 kDa were investigated by MS and MS/MS analysis. The gel image was processed with the Adobe Photoshop program.

### 2.5. Proteomic Analysis of OMVs and Protein Extraction

The proteins from OMVs were identified using a classical gel-based proteomic approach. Briefly, the resulting bands were under the trypsin-catalysed in-gel digestion procedure. NanoUPLC-hrMS/MS analyses of the resulting peptide mixtures were performed on a Q-Exactive orbitrap mass spectrometer (Thermo Fisher Scientific, Waltham, MA, USA), coupled with a nanoUltimate300 UHPLC system (Thermo Fisher Scientific). Peptide separation was conducted on a capillary EASY-Spray PepMap column (0.075 mm × 50 mm, 2 µm, Thermo Fisher Scientific) using aqueous 0.1% formic acid (A) and CH3CN containing 0.1% formic acid (B) as mobile phases and a linear gradient from 3% to 40% of B in 45 min and at a 300 nL·min^−1^ flow rate. Mass spectra were acquired over an m/z range from 400 to 1800. To achieve protein identification, MS and MS/MS data were analysed via Mascot software (v2.5, Matrix Science, Boston, MA, USA) analysis, using the non-redundant Data Bank UniprotKB/Swiss-Prot (Release 2020_03). Parameter sets were: (i) trypsin cleavage; (ii) carbamidomethylation of cysteine as a fixed modification and methionine oxidation as a variable modification; (iii) a maximum of two missed cleavages; and (iv) false discovery rate (FDR), calculated by searching the decoy database, ≤0.05.

### 2.6. Cellular Exposure to OMVs

BEAS 2B cells (5 × 10^5^ cells per well) were plated in a 6-well tissue culture plate at 37 °C for 24 h. After 24 h, the culture medium was removed and OMVs from three strains (5 μg/mL) were added. As a negative control, the same volume of OMV solvent (PBS1X) was added to the cells. Six hours post exposure, cells were collected, and miRNA extractions were performed.

### 2.7. RNA Extraction and miRNome Profiling

Total RNA was purified with the PARIS mirVANA kit (Invitrogen, Carlsbad, CA, USA), according to the manufacturer’s protocol. The RNA concentration was examined by NanoDrop 1000 spectrophotometer (Thermo Fisher Scientific). RNA reverse transcription was performed using the TaqMan MiRNA reverse transcription kit and Megaplex RT primers (Thermo Fisher Scientific). The expression of microRNAs in BEAB 2B cells was determined using the TaqMan Human MicroRNA array (Thermo Fisher Scientific). Briefly, the single-stranded cDNAs were amplified using the TaqMan Universal Master Mix PCR, and specific primers and probes present on the 384-well TaqMan miRNA array card (Thermo Fisher Scientific). The array was conducted on the Applied Biosystems Viia7 instrument (Life Technologies, Carlsbad, CA, USA) with the predefined thermal cycle conditions.

### 2.8. Statistical Analyses

To normalize the three miRNA data sets, the raw data of the cycle threshold (Ct) were processed in R, excluding the undetectable data, i.e., with a value of C*t* > 34. The ΔC*t* values were obtained by subtracting the average of the C*t* values of the internal endogenous controls (RNU44, RNU48 and U6) from the C*t* value of the miRNA for the given sample, C*t*0. The ΔΔC*t* was acquired by subtracting the ΔC*t* of the sample from the sample control. The expression fold change was calculated by increasing the power of the negative ΔΔC*t* value by 2. The relationship between C*t*, ΔC*t*, ΔΔC*t* and fold change (FC) is given by the following equation: ΔC*t* = C*t* − C*t*0; ΔΔC*t* = ΔC*t* − ΔC*t*control; FC = 2^−ΔΔC*t*^. We performed a t-test to identify the de-regulated miRNAs. A *p*-value <= 0.005 indicated a significant difference. In particular, miRNAs were upregulated for logFC ≥ 1.5 while they were downregulated for logFC ≤ −0.5. After normalising, a heat map of the data was generated using the MeV software in order to produce an informative visualisation through the MeV software (MeV v4.9.0, Shanghai, China). Complete linkage clustering with the Manhattan distance measurement method for the three datasets was used.

### 2.9. Prediction and Function of miRNA Target Genes

Three different databases, TargetScan (TargetScan v7.1, Cambridge, MA, USA), DIANA-microT-CDS (DIANA v5.0, Lamia, Greece) and miRTarBase (miRTarBase v8.0 beta, Hsinchu, Taiwan) were exploited to predict the target genes of the miRNA dataset. In agreement with the parameters set for each bioinformatic tool, the genes commonly predicted by all three algorithms were selected. The target genes were represented by the VennDiagram package in R. The Metascape software (Metascape v5.0, San Diego, CA, USA) was used for gene ontology (GO) enrichment analysis. The output of the analysis was associated with a *p* < 0.005. In addition, the Metascape bioinformatics tool was used to establish the protein–protein interaction networks (PPI-Nets) and topological structure analysis was performed.

### 2.10. miRNA Expression Analysis

After total RNA extraction, miRNA sequences were converted into cDNA using the miScript II RT Kit (Qiagen), according to the manufacturer’s protocol. Real-time quantitative PCR (RT-qPCR) was carried out with QuantiTectSYBR Green PCR Kit (Qiagen). The thermal protocol was as follows: 95 °C for 15 min plus 40 cycles at 94 °C for 30 s, 58 °C for 34 s and 70 °C for 34 s [44]. RT-qPCR data were the result of three independent experiments, each with three replicates and were represented as ± s.e.m. RNA U6 small nuclear 6 pseudogene (RNU6-6P) was used for data normalization. The miRNA primers used in RT-qPCR are reported in Table 1.

## 3. Results

### 3.1. Characterization of K. pneumoniae-Derived OMVs

In order to define the structural and functional characteristics of OMVs produced by *K. pneumoniae*, vesicles were purified from three different strains: *K. pneumoniae* ATCC 10031, MS *K. pneumoniae* (clinical isolate) and KPC-producing *K. pneumoniae* (clinical isolate). The strains were cultured to stationary phase and their OMVs were collected. To guarantee precise accuracy of the analyses, three independent purifications of the OMVs were performed for each strain. All vesicles were analysed in terms of diameter and size distribution, through DLS. DLS analysis showed that most OMVs of *K. pneumoniae* ATCC 10031 presented a diameter of 273.3 ± 1.3 nm and were characterized by a slightly heterogeneous size distribution, confirmed by the polydispersity index of 0.329 ± 0.021. OMV vesicles from isolated clinical strains showed an increase in size and a greater heterogeneity of vesicular populations. The OMVs of MS *K. pneumoniae* predominately exhibited a diameter of 427.1 ± 0.9 nm and the vesicle population showed a high heterogeneity, demonstrated by a polydispersity index of 0.417 ± 0.017. Similar results were obtained for OMVs produced by KPC-producing *K. pneumoniae*. The majority of these vesicles presented with a diameter of 483.3 ± 1.7 nm and a polydispersity index of 0.333 ± 0.132, suggesting a heterogeneous population in size distribution (Table 2). All purified OMVs were quantified based on protein yield. Protein concentrations of 0.08 ± 0.06 mg/mL, 0.14 ± 0.03 mg/mL and 0.21 ± 0.01 mg/mL had been generated by *K. pneumoniae* ATCC 10031, MS *K. pneumoniae* and KPC-producing *K. pneumoniae*, respectively, for 600 mL of LB culture (Table 3).

### 3.2. SDS-PAGE and LC-MS/MS Analysis of OMVs

**Figure 1:**
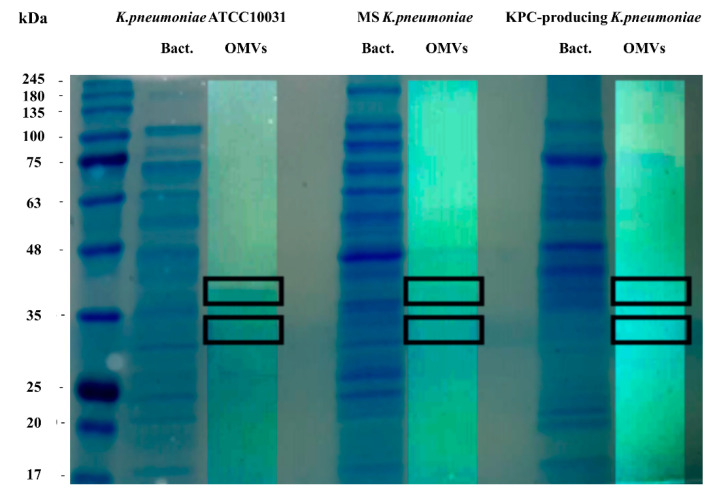
Coomassie-stained SDS-PAGE (10%) protein profiles of *K. pneumoniae* ATCC 10031, MS *K. pneumoniae* and KPC-producing *K. pneumoniae* and relative OMVs. Molecular mass marker (MW) is expressed in kilodaltons (kDa). The rectangles indicate the bands subjected to trypsin digestion and MS and MS/MS analysis.

To evaluate the protein profile of the OMVs purified from *K. pneumoniae* ATCC 10031, MS *K. pneumoniae* and KPC-producing *K. pneumoniae*, 3.3 μg of protein was subjected to 10% SDS-PAGE (Figure 1). Two major bands, in the range of 30–40 KDa, were detected in the OMVs from *K. pneumoniae* ATCC 10031, MS *K. pneumoniae* and KPC-producing *K. pneumoniae*, with a clear difference from the bacterial lysate protein profile, confirming the absence of bacterial contaminants. The main protein bands were digested with trypsin and mass spectrometry-based proteomic analysis was performed. Mass spectra analysis identified eight proteins common to all purified OMVs. The list of OMV proteins is reported in Table 4 in which identification name, function, molecular weight, and the total score values are indicated.

### 3.3. K. pneumoniae-Derived OMVs Affect miRNA Expression Profile in BEAS-2B Cells

**Figure 2:**
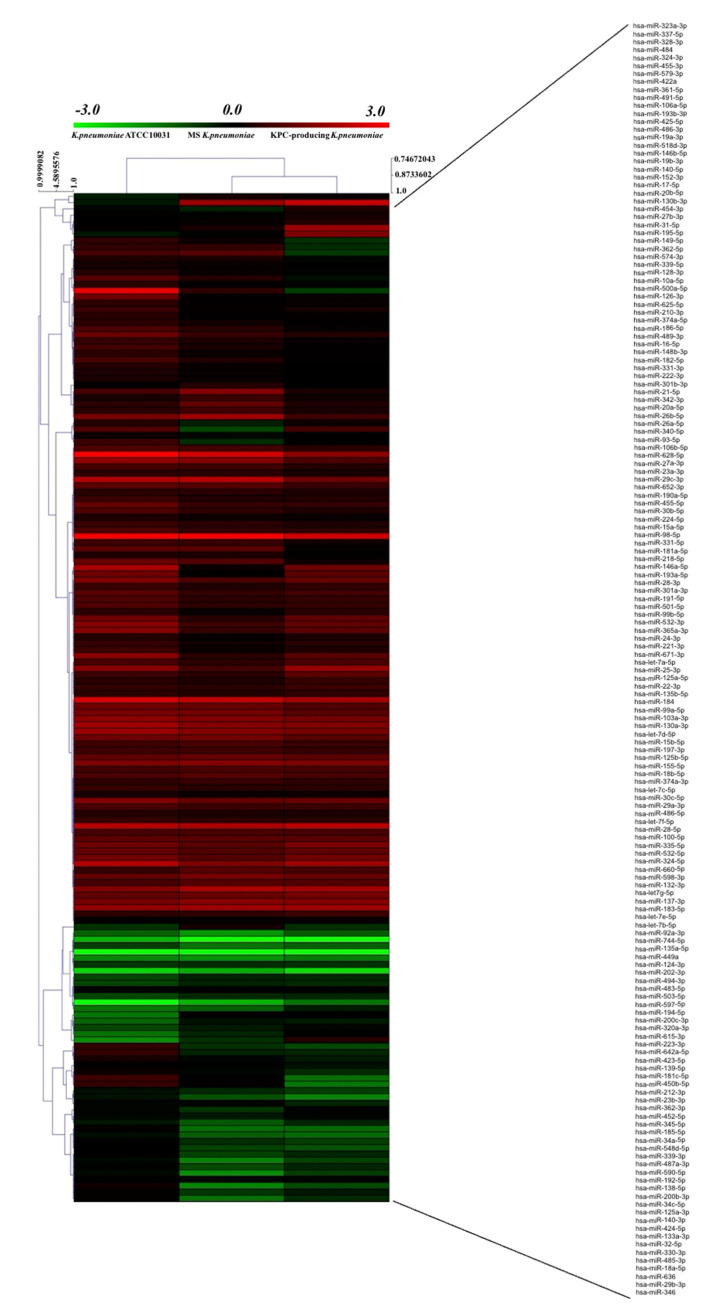
Heat map and hierarchical analysis of clusters. Heat map based on microarray results filtered and processed by bioinformatics analysis. Red indicates a greater expression than the control and green indicates a lower expression.

**Figure 3:**
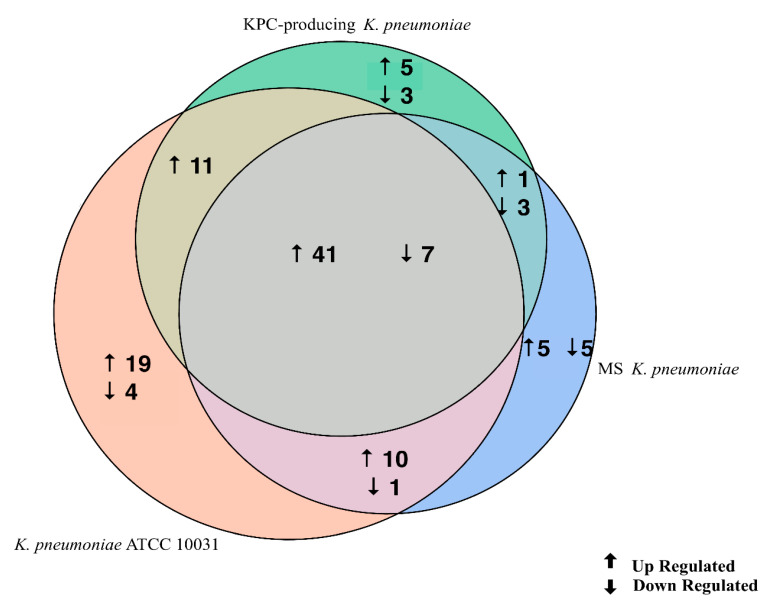
Venn diagram of differentially expressed miRNAs in three samples. The Venn diagram shows the different expressions of miRNAs after exposure of BEAS-2B cells to OMVs purified from three different strains of *K. pneumoniae* (ATCC 10031, MS and KPC-producing) compared to the control sample. The numbers in the intersecting circles represent the miRNA sequences commonly dysregulated in the different samples.

The evaluation of the expression profiles of miRNAs was carried out after treating BEAS 2B cells with OMVs purified from *K. pneumoniae* ATCC 10031, MS *K. pneumoniae* and KPC-producing *K. pneumoniae*. Using the TaqMan miRNA Array CARD, we screened the expression level of 384 miRNA sequences [45]. Raw microarray data were filtered and analysed. To give an illustrative and informative depiction, the results are shown via heatmap, using MeV software (MultiExperiment Viewer) (Figure 2). Transcripts with upregulated expression are indicated in red, while downregulated transcripts are indicated in green. In particular, the analysis revealed 115 miRNA sequences that were differentially regulated in treated samples compared to untreated controls (*p* < 0.05; cut-off > 1.5 or < −0.5). *K. pneumoniae* ATCC 10031 derived OMVs induced the upregulation of 81 and downregulation of 13 miRNAs. In cells treated with MS *K. pneumoniae* derived OMVs, 57 miRNAs were upregulated and 16 were downregulated. Incubation with KPC-producing *K. pneumoniae* derived OMVs altered the expression of 71 miRNAs (58 upregulated and 13 downregulated). The differential analysis of miRNome profiling in response to each of the three treatments was compared and shown in the Venn diagram in Figure 3. The dysregulated miRNAs were common to all the three samples and the individually dysregulated miRNAs in each sample were used for gene ontology, biological function, and pathway analysis.

### 3.4. Functional Characterization of Target Genes

**Figure 4:**
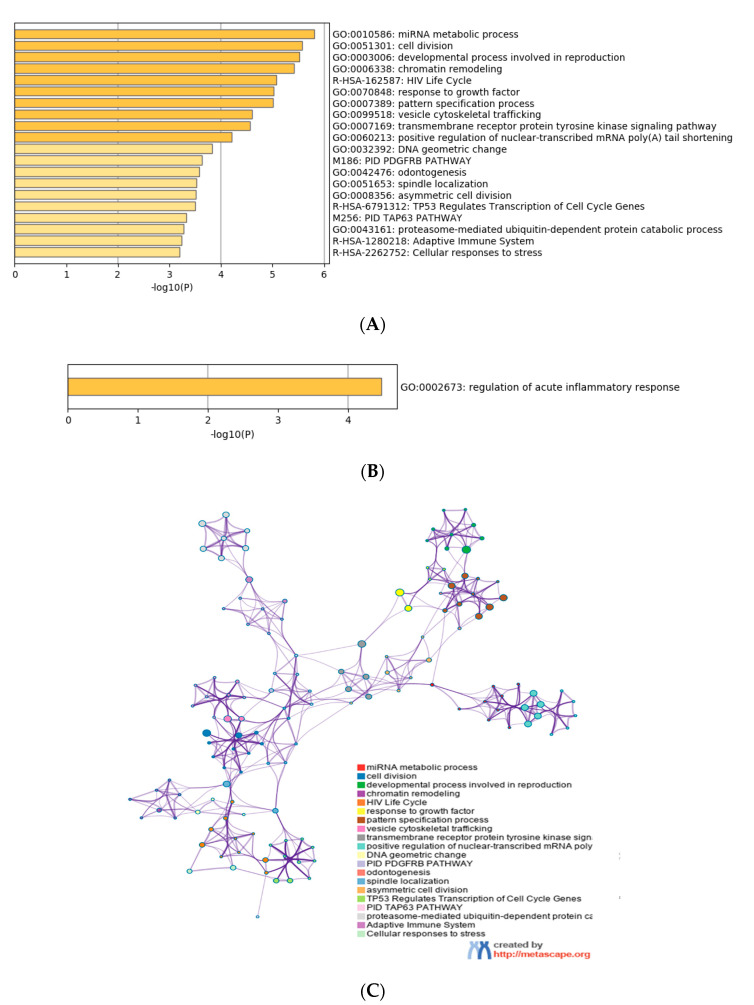
Analysis of the functional enrichment of target genes. (**A**,**B**) The main enrichment analysis clusters detected by Metascape of genes associated with upregulated miRNA after treatment with OMVs; (**C**) interaction network of the clusters detected by Metascape. The nodes of the same colour belong to the same cluster. Terms with a similarity score > 0.3 are linked by an edge. The network is visualized with Cytoscape (v3.1.2) with a “force-directed” layout and edge bundled for clarity.

**Figure 5:**
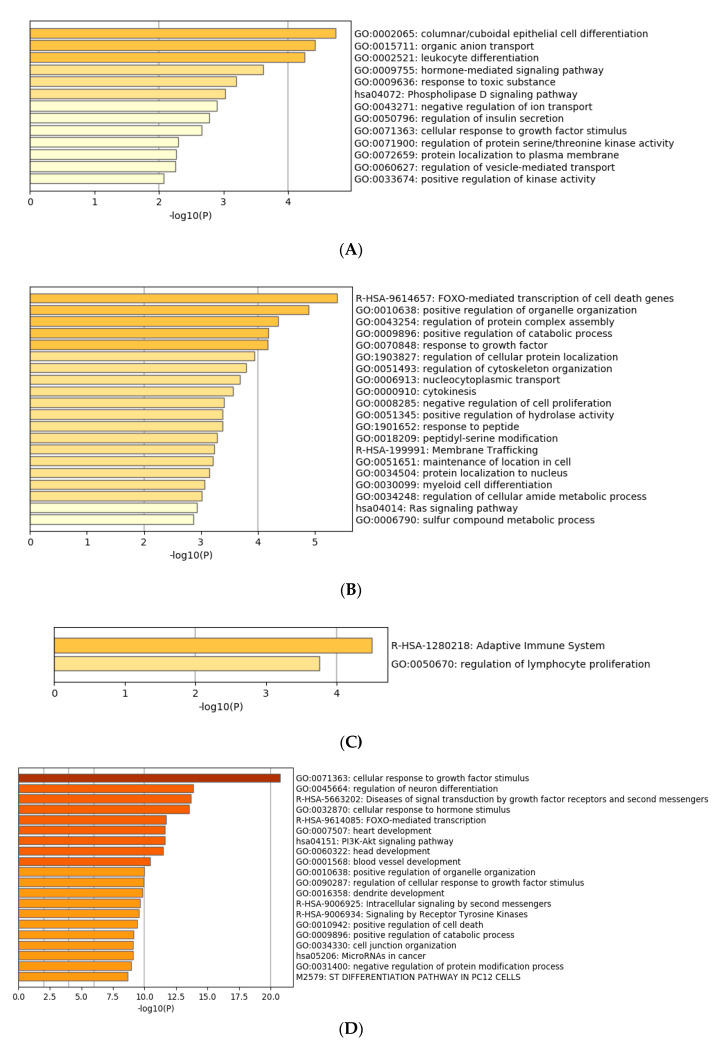

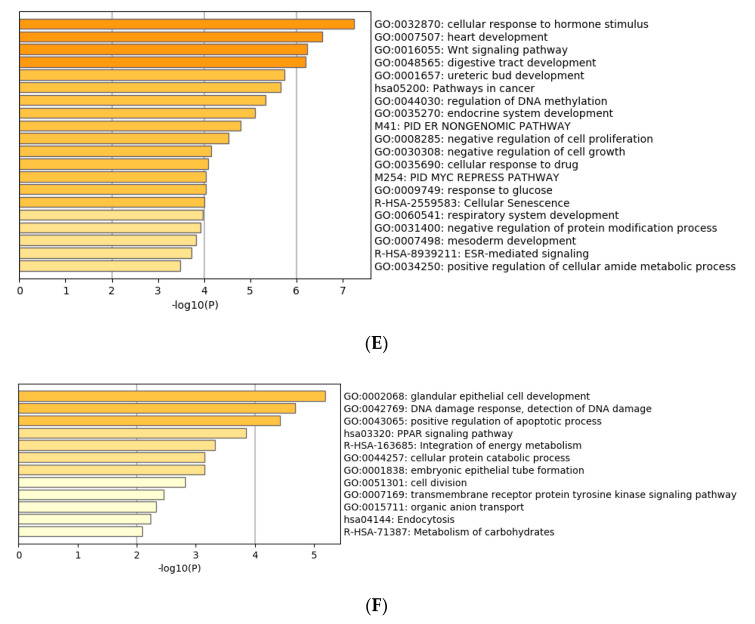
Gene prediction analysis. MiRNA sequences up and downregulated in the BEAS-2B sample exposed to OMVs of *K. pneumoniae* ATCC 10031 (**A**,**B**), MS *K. pneumoniae* (**C**,**D**) and KPC-producing *K. pneumoniae* (**E**,**F**).

Gene ontology enrichment analysis was performed using Metascape software. The DIANA gene, Target Scan gene and MirTarBase gene software were exploited to filter predicted data. There were 41 miRNAs that were upregulated and seven that were downregulated in all samples following exposure to OMVs from three different *K. pneumoniae* strains. Predicted target genes of upregulated miRNA sequences in BEAS-2B cells were significantly associated with “miRNA metabolic processes” (GO: 0010586), “cell division” (GO: 0051301), “developmental processes involved in reproduction” (GO: 0003006), “chromatin remodelling” (GO: 0006338) and “response to growth factor” (GO: 0070848) (Figure 4A). Target genes of seven downregulated miRNA sequences were involved in “regulation of acute inflammatory response” (GO: 0002673) (Figure 4B). All identified biological processes are closely related (Figure 4C). Our analysis also assessed the differences in expression of miRNA induced by *K. pneumoniae* ATCC 10031, MS *K. pneumoniae* and KPC-producing *K. pneumoniae* OMVs. After exposure with *K. pneumoniae* ATCC 10031, 19 and four miRNA sequences are upregulated and downregulated, respectively. Upregulated miRNA sequences were involved in “glandular epithelial cell development” (GO: 0002068), “DNA damage response, detection of DNA damage” (GO:0042769) and “positive regulation of apoptotic process” (GO: 0043065) (Figure 5A). The downregulated miRNAs were strongly associated with “cellular response to hormone stimulus” (GO: 0032870) (Figure 5B). The treatment with the MS strain upregulated and downregulated five different miRNA sequences in BEAS-2B cells. Five upregulated miRNA sequences were involved with the “adaptive immune system” (R-HSA-1280218) (Figure 5C) while the five downregulated miRNA sequences were related to the “cellular response to growth factor stimulus” (GO: 0071363), “regulation of neuron differentiation” (GO: 0045664) and “disease of signal transduction by growth factor receptors and second messengers “(R-HSA-5663202) (Figure 5D). The exposure to KPC-producing *K. pneumoniae* OMVs resulted in the upregulation and downregulation of five and three miRNA sequences, respectively. Upregulated sequences were involved in “FOXO-mediated transcription of cell death genes” (R-HSA-9614657), “positive regulation of organelle organization” (GO: 0010638) and “regulation of protein complex assembly” (GO: 0043254) (Figure 5E). Downregulated miRNA sequences were mainly associated with “columnar/cuboidal epithelial cell differentiation” (GO: 0002065) (Figure 5F).

### 3.5. miRNAs Validation

**Figure 6:**
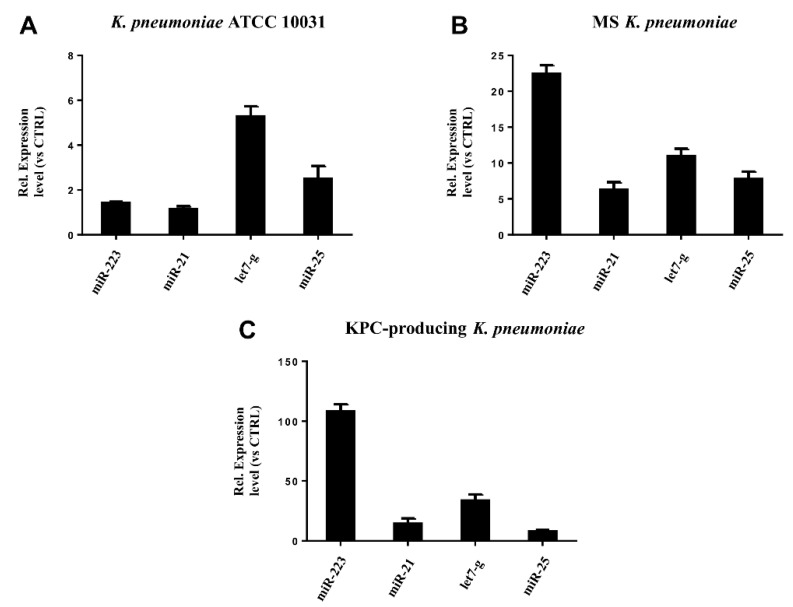
Validation of miRNA microarray data by real-time PCR. The expression levels of miR-21, miR-25, miR223, and let-7g were consistent with the miRNA microarray results after treatment with *K. pneumoniae* ATCC 10031 (**A**), MS *K. pneumoniae* (**B**) and KPC-producing *K. pneumoniae* (**C**).

RT-qPCR was used to confirm the gene expression results obtained from microarray analysis. Four miRNAs (hsa-miR-223, hsa-miR-21, hsa-miR-25, hsa-let-7g) were selected for validation. The expression of the analysed miRNAs showed a good compliance with microarray data (Figure 6). These findings suggest that the microarray data were reliable, supported by similar fold changes. The expression levels in the miRNAs hsa-miR-223, hsa-miR-21 and hsa-let-7g were significantly higher in the treatment with KPC-producing *K. pneumoniae* OMVs. In contrast, the OMVs from MS strain induced higher expression levels than OMVs from ATCC 10031 strain. For hsa-miR-25, there were no significant variations in expression between treatment with OMVs derived from KPC-producing *K. pneumoniae* and MS strain.

## 4. Discussion

*K. pneumoniae* represents a worrying multi-resistant bacterium that causes nosocomial infections with high morbidity and mortality rates. A better understanding of the pathogenesis of *K. pneumoniae* infections is crucial. Several studies have shown that *K. pneumoniae* produces and secretes OMVs, which represent an important vehicle to transport many virulence effectors to host cells [46,47,48]. To the best of our knowledge, this was the first study reporting the differential expression of specific miRNA and their predicted target genes after exposure with OMVs purified from *K. pneumoniae* ATCC 10031, MS *K. pneumoniae* and KPC-producing *K. pneumoniae*. In this study, we highlight the different features of OMVs derived from three *K. pneumoniae* strains. The size of the vesicles was larger in clinical isolates (MS and KPC-producing) compared to the reference strain. These findings suggest that an increased load of virulence determinants could probably occur in the vesicles from clinical isolates. SDS-PAGE analysis of OMVs purified from *K. pneumoniae* ATCC 10031, MS *K. pneumoniae* and KPC-producing *K. pneumoniae* revealed two main protein bands in a range from 30 to 40 kDa. MS and MS/MS study identified these protein bands as outer membrane protein A, outer membrane porin C, glyceraldehyde-3-phosphate dehydrogenase, malate dehydrogenase, glucokinase, 2-dehydro-3-deoxyphosphooctonate aldolase, aminomethyltransferase, L-threonine 3-dehydrogenase and elongation factor Ts. The evident difference in the protein profile of the bacterial lysate suggests that the purification protocol exploited to isolate vesicles was successful in separating OMVs from bacterial contaminants.

Currently, no study has evaluated changes in cellular miRNoma after exposure to *K. pneumoniae* OMVs. In the present study, human bronchial epithelial cells were exposed to OMVs from *K. pneumoniae* ATCC 10031, MS *K. pneumoniae* and KPC-producing *K. pneumoniae* and cellular miRNoma was evaluated. Microarray analysis showed 115 differentially expressed miRNA sequences compared to the untreated sample. In particular, OMVs derived from *K. pneumoniae* ATCC 10031 caused upregulation and downregulation of 81 and 13 miRNAs, respectively. OMVs purified from MS *K. pneumoniae* changed the expression of 73 miRNAs (57 upregulated and 16 downregulated). Moreover, OMV treatment from KPC-producing *K. pneumoniae* induced the upregulation of 58 miRNAs and the downregulation of 13 miRNAs. Only 48 miRNA sequences were commonly dysregulated after treatment with the different vesicles. Of the latter, miR-223, hsa-miR-21, hsa-miR-25 and hsa-let-7g sequences were validated using RT-qPCR. MiR-223 has shown strong upregulation in the treatment with OMVs derived from KPC-producing *K. pneumoniae*. This miRNA has previously been shown to increase production of NF-κB mediated inflammatory cytokines [49]. In particular, miR-223 regulates IL-6 production, aiming to focus on STAT3 to improve TLR-mediated inflammatory responses [50]. Several studies have suggested that miR-223 is an important regulator of the innate immune system and response to bacterial stimulation. Levels of miR-223 were shown to gradually increase, doubling in 6 h, then showing a three-fold increase after 24 h in *Helicobacter pylori*-infected THP-1 monocytes [51]. LPS is also able to regulate the expression of miR-223. Overexpression of miR-223 sequences occurs in LPS-activated macrophages [52]. Similar to the miR-223 sequence, hsa-miR-21 also acts in the control of the inflammatory response. This sequence was upregulated in all of the three treatments. By silencing the programmed cell death 4 (PDCD4) gene, hsa-miR-21 increased the production of the anti-inflammatory cytokine IL-10 and reduced NF-κB-induced inflammatory activity [53]. The high hsa-miR-21 expression was previously found in periodontal ligament tissues of patients with periodontitis. *Porphyromonas gingivalis* LPS exposure induced overexpression of miR-21 sequences in a murine macrophage cell line [54]. Moreover, in *H. pylori*-infected gastric epithelial cells, upregulation of MiR-21 occurred. [55]. Hsa-miR-25 is closely involved in the inflammatory response, by modulating changes in cytokine levels. This sequence can inhibit the expression of suppressor of cytokine signalling (SOCS) genes after cell treatment with *K. pneumoniae* OMVs [56]. Hsieh et al. showed that C57BL/6 mice receiving intraperitoneal injections of LPS from several bacterial species (*E. coli*, *K. pneumoniae*, *P. aeruginosa*, *Salmonella enterica* and *Serratia marcescens*) induced upregulated expression of miR-25 sequences in blood and epithelial cells [51]. Only hsa-let-7g showed significant downregulation, following treatment with OMVs purified from the three strains of *K. pneumoniae*. This miRNA sequence negatively regulates IL-6 expression, after LPS exposure [44]. *S. enterica* and its LPS component induced downregulation of let-7g in macrophages and epithelial cells, promoting the expression of cytokines [57]. These findings suggest that the downregulation of let-7g in BEAS-2B could be mostly attributed to the LPS component of OMVs.

Our present research study shows that the dysregulation of the selected miRNA suggests that OMVs derived from three *K. pneumoniae* strains represent potent inflammatory agents. In concordance with previously published studies, we confirmed that OMVs derived from *K. pneumoniae* contribute to the pathogenesis of infection in the host. Additionally, our findings improve the current understanding about the size and composition of OMVs, elucidating miRNA-mediated mechanisms related to *K. pneumoniae* infection. We are tempted to speculate about further investigations directed towards the development of OMVs as innovative vaccine strategies.

## Figures and Tables

**Table 1:** Qiagen GeneGlobe IDs of microRNA (miRNA) target sequences.

Target Name	GeneGlobe ID
RNU6-6P	MS00033740
hsa-miR-223	MS00009184
hsa-miR-21	MS00009079
hsa-miR-25	MS00003227
hsa-let-7g	MS00008337

**Table 2:** Dynamic light scattering (DLS) analysis measurements of the Z-average size (Z-ave) and polydispersity index (PDI) of the outer membrane vesicles (OMVs).

Bacterial Strain	Z-Ave (d.nm)	PDI
*K. pneumoniae* ATCC 10031	273.3 ± 1.3	0.329 ± 0.021
MS *K. pneumoniae*	427.1 ± 0.9	0.417 ± 0.017
KPC-producing *K. pneumoniae*	483.3 ± 1.7	0.333 ± 0.132

**Table 3:** Protein concentration of OMVs purified from different strains of *K. pneumoniae.*

Bacterial Strain	Protein Concentration [mg/mL]
*K. pneumoniae* ATCC 10031	0.08 ± 0.06
MS *K. pneumoniae*	0.14 ± 0.03
KPC-producing *K. pneumoniae*	0.21 ± 0.01

**Table 4:** Protein profile commonly present in OMVs purified from different *K. pneumoniae* strains.

Protein	Function	Theoretical MW (Da)	Score
Outer membrane protein A	Action of colicins K and L	37,152	1501
Outer membrane porin C	Passive diffusion across the outer membrane	39,639	1298
Glyceraldehyde-3-phosphate dehydrogenase (Fragment)	Enzyme in glycolysis	32,457	680
Nucleoside-specific channel-forming protein	Receptor for colicin K	33,486	348
Malate dehydrogenase	Enzyme in Krebs cycle	32,549	180
Glucokinase	Enzyme in glycolysis	34,756	106
2-dehydro-3-deoxyphosphooctonate aldolase	Enzyme in aminoacids synthesis	31,033	97
Aminomethyltransferase	enzyme in the glycine cleavage complex	39,904	88
L-threonine 3-dehydrogenase	Enzyme in L-threonine catabolism	37,559	33
Elongation factor Ts	Protein biosynthesis	30,545	18
